# MoS_2_ with Controlled Thickness for Electrocatalytic Hydrogen Evolution

**DOI:** 10.1186/s11671-021-03596-x

**Published:** 2021-08-31

**Authors:** Xiaoxuan Xu, Lei Liu

**Affiliations:** 1 Nanjing Vocational University of Industry Technology , Nanjing, 210023 People’s Republic of China; 2 School of Mechanical Engineering, Southeast University, Nanjing, 211189 People’s Republic of China

**Keywords:** MoS_2_, Atomic layer deposition, Hydrogen evolution, MoS_2_ thickness

## Abstract

Molybdenum disulfide (MoS_2_) has moderate hydrogen adsorption free energy, making it an excellent alternative to replace noble metals as hydrogen evolution reaction (HER) catalysts. The thickness of MoS_2_ can affect its energy band structure and interface engineering, which are the avenue way to adjust HER performance. In this work, MoS_2_ films with different thicknesses were directly grown on the glassy carbon (GC) substrate by atomic layer deposition (ALD). The thickness of the MoS_2_ films can be precisely controlled by regulating the number of ALD cycles. The prepared MoS_2_/GC was directly used as the HER catalyst without a binder. The experimental results show that MoS_2_ with 200-ALD cycles (the thickness of 14.9 nm) has the best HER performance. Excessive thickness of MoS_2_ films not only lead to the aggregation of dense MoS_2_ nanosheets, resulting in reduction of active sites, but also lead to the increase of electrical resistance, reducing the electron transfer rate. MoS_2_ grown layer by layer on the substrate by ALD technology also significantly improves the bonding force between MoS_2_ and the substrate, showing excellent HER stability.

## Introduction

Hydrogen energy has become an excellent choice for solving global energy shortages and environmental pollution due to its own advantages (such as abundant sources, high energy density, and only water as combustion products) [1–3]. Hydrogen production by electrolysis of water is considered to be a green hydrogen production technology because it can get rid of the dependence on carbon-containing fossil fuels [4, 5]. Although the hydrogen evolution reaction (HER) can produce hydrogen, its high energy consumption and low yield have always been a concern [6]. Platinum (Pt)-based noble metal catalysts have shown strong catalytic activity, but their higher prices and lower reserves have prevented them from being applied in industry [7]. Therefore, exploring and developing non-noble metal catalysts with abundant reserves, low price, high efficiency and durability is an important strategy to promote the application of hydrogen energy, which has become one of the most important research hotspots [8–10].

At present, transition metal oxides, sulfides, phosphides, nitrides, carbides, alloys and other catalysts have been developed for HER [11–15]. Among them, molybdenum disulfide (MoS_2_) has an activity close to that of Pt in catalytic sites and it becomes a preferred Pt substitute material in non-noble metal chalcogenides theoretically [16]. Unlike the bulk phase, the two-dimensional (2-D) MoS_2_ with layered structure exhibits unique surface effects, small size effects, and macroscopic quantum tunneling effects, which greatly improves related HER performance [17, 18]. However, the 2-D MoS_2_ is prone to stacking, which reduces the number of edge active sites and affects hydrogen production [19]. In order to make full use of the active sites of MoS_2_, a few layers of MoS_2_ are attempted to manufacture. The commonly preparation methods mainly include the “top-down” method represented by the micromechanical force stripping, the lithium ion intercalation, the liquid phase ultrasonic method, and the “bottom-up” method represented by the high temperature thermal decomposition, vapor deposition, hydrothermal method [20–22]. Among them, “top-down” is difficult to achieve high-efficiency reproducible manufacturing and the “bottom-up” is relatively controllable and has a wide range of applications. Chemical vapor deposition (CVD) is a representative method in manufacturing fewer layers of MoS_2_ films [23]. Although the MoS_2_ films prepared by CVD exhibit high quality, such as a flat surface, less lattice distortion and other defects, CVD cannot uniformly produce MoS_2_ on the surface of a structure with a high aspect ratio [24]. In addition, because of low stability and low repeatability, the CVD method cannot be used to manufacture MoS_2_ with a large scale.

As a specially modified CVD method, atomic layer deposition (ALD) is also used to fabricate thin film materials [25]. In an ALD cycle, through a self-limiting chemical reaction, a complete reaction is interrupted into two half-reactions [26]. Only when the active sites of surface are exhausted, the first half reaction stops, and then another half reaction will proceed [27]. The chemical reaction of the newly fabricated atomic film is directly determined by the previous layer, so only one layer of atoms can be deposited per ALD cycle [28]. During the ALD process, not only the thickness of the film can be precisely controlled, but the uniformity of the film on the substrate with complex morphology can also be well maintained [29]. In addition, because the manufacturing process is not sensitive to the amount of precursor, ALD has high repeatability. Therefore, ALD is suitable for the controlled manufacture 2-D MoS_2_ films [30].

In this work, MoS_2_ with different thicknesses were controllably grown on glassy carbon (GC) substrates through ALD technology, and it was directly used as a catalyst for HER without binders. The hydrogen evolution performance of MoS_2_/GC in acid solution was studied, and the related mechanism was also analysed.

## Methods

The current study was aimed to improve the HER performance of MoS_2_ by adjusting its thickness.

### Materials

Glassy carbon (GC, 15 mm × 10 mm × 1 mm) was purchased from Beijing Anatech Co., Ltd. Molybdenum pentachloride (MoCl_5_, 99.6%) was purchased from Shanghai Aladdin Bio-Chem Technology Co., Ltd. Hydrogen sulfide (H_2_S, 99.6%) and Nitrogen (N_2_, 99.999%) were received from Nanjing Special Gas Factory Co., Ltd.

### Preparation of MoS_2_ on GC

GC with excellent conductivity was used as the substrate for manufacturing the few layers MoS_2_ film. The GC was ultrasonically cleaned with acetone, ethanol and deionized water for 5 min, and then treated with plasma for 5 min. The MoS_2_ film was deposited on GC using a commercial ALD equipment (Sunaletmr-100, Picosun). Before depositing process, the reaction chamber and Mo source were heated to 460 °C and 210 °C, respectively, and stabilize for one hour. Then, MoCl_5_ and H_2_S were alternately injected into the reaction chamber. The carrier gas used was N_2_ and the flow rate was 50 sccm. The pulse time for source and cleaning was 0.5 s, 30 s, 0.5 s and 30 s, respectively. By controlling the number of ALD cycles to 50, 100, 150, 200 and 250, the preparation of MoS_2_ films with different thicknesses were achieved.

### Characterization

Scanning electron microscope (SEM) was used to observe the morphology of the catalyst by Inspect-F50 (FEI) instrument, and the acceleration voltage was 20 kV. High resolution-transmission electron microscope (HR-TEM) images were obtained on JEM-2100 (Olympus) instrument, and the acceleration voltage was 200 kV. X-ray diffraction (XRD) was employed to study the crystal phase structure by Smartlab-3 (Rigaku). Raman spectrometer (Raman) was used for analysis of solid surface composition by XperRam C (Nanobase) instrument, and the excitation wavelength is 532 nm. Atomic force microscopy (AFM, D-5A, Micronano) was used to test the morphology and thickness of the MoS_2_ film.

### Electrochemical Tests

All electrochemical measurements were tested on a CHI660E electrochemical workstation (CH Instruments). Electrochemical measurements were performed in three electrode system. The counter electrode, reference electrode and working electrode are carbon rod, Ag/AgCl and MoS_2_/GC respectively. The hydrogen production polarization curve adopts linear sweep voltammetry (LSV), the sweep rate is 5 mV/s, the sweep range is − 0.5 to 0 V, and the electrolyte is 0.5 M H_2_SO_4_. None of the LSV curves were iR corrected. Through the Nernst equation, All the electrochemical potentials were converted into Reversible hydrogen electrode (RHE) voltages: *E* (RHE) = *E* (Ag/AgCl) + 0.159 V. The frequency ranges of electrochemical impedance spectroscopy (EIS) is 1 Hz–100 kHz, and the overpotential is 200 mV. The cyclic voltammetry (CV) and chronoamperometry (i-t) were used to estimate the stability. The electrochemical double layer capacitance (*C*_dl_) test adopted the CV curve under different scanning rates. The CV test voltage range was 0.1–0.2 V (vs. RHE), scan rate was 20–140 mV/s. The electrochemically active surface area (ECSA) was calculated from the specific current density through the following relationship:

$${{A}}_{\text{ECSA}}=\frac{\text{specific~capacitance}}{\text{40}~\upmu{\text{F}}\cdot{\text{cm}}^{-2}~ {\text{percm}}_{\text{ECSA}}^{2}}$$

## Results and Discussion

**Fig. 1:**
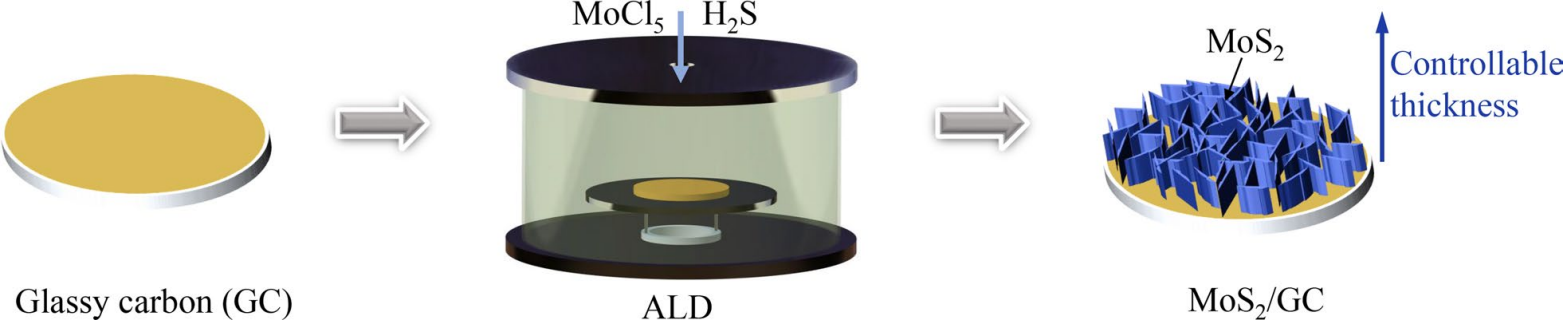
Schematic representation of the controlled synthesis of MoS_2_ by ALD

As shown in Fig. 1, MoS_2_ films with different thicknesses were prepared on the GC substrate by the ALD with MoCl_5_ and H_2_S as precursors under 460 °C. The MoS_2_ films prepared at 50, 100, 150, 200 and 250 ALD cycles were named 50ALD-MoS_2_/GC, 100ALD-MoS_2_/GC, 150ALD-MoS_2_/GC, 200ALD-MoS_2_/GC and 250ALD-MoS_2_/ GC respectively. MoS_2_/GC can be used directly as a catalytic electrode without the need to load the catalyst on other electrodes through a binder (Nafion), which is more conducive to the large-scale manufacture and practical application.

**Fig. 2:**
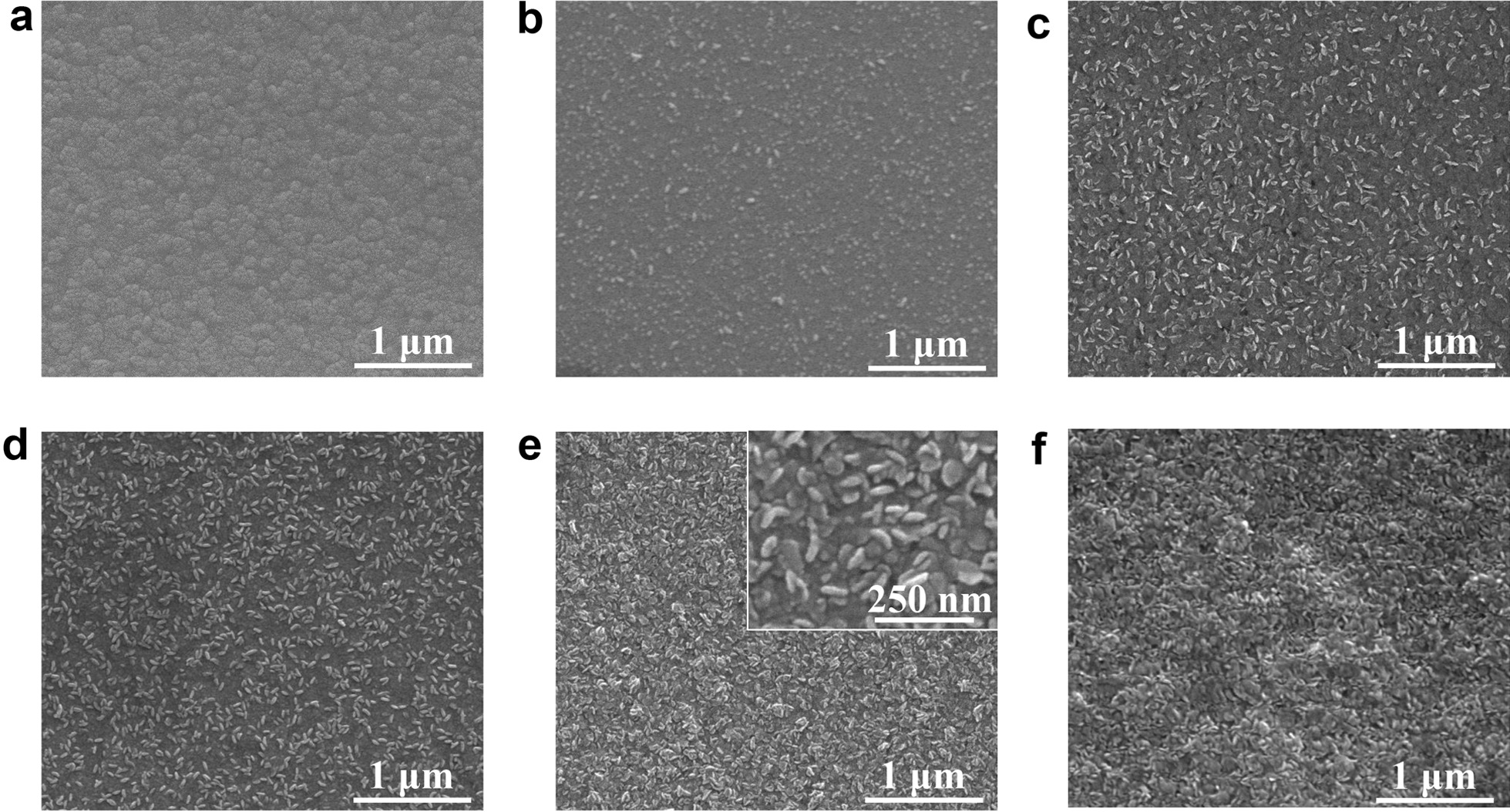
SEM images of **a** GC, **b** 50ALD-MoS_2_/GC, **c** 100ALD-MoS_2_/GC, **d** 150ALD-MoS_2_/GC, **e** 200ALD-MoS_2_/GC and **f** 250ALD-MoS_2_/GC

From the SEM images (Fig. 2), it can be seen that the MoS_2_ films prepared by ALD on the GC substrate has good coverage and consistency. As the number of cycles increases, the MoS_2_ films gradually become thicker, and the aggregation states change from nanoparticles to larger nanosheets. When the ALD cycle is low, MoS_2_ grows in a direction parallel to the substrate, and when the number of cycles increases, MoS_2_ grows vertically to form nanosheets.

**Fig. 3:**
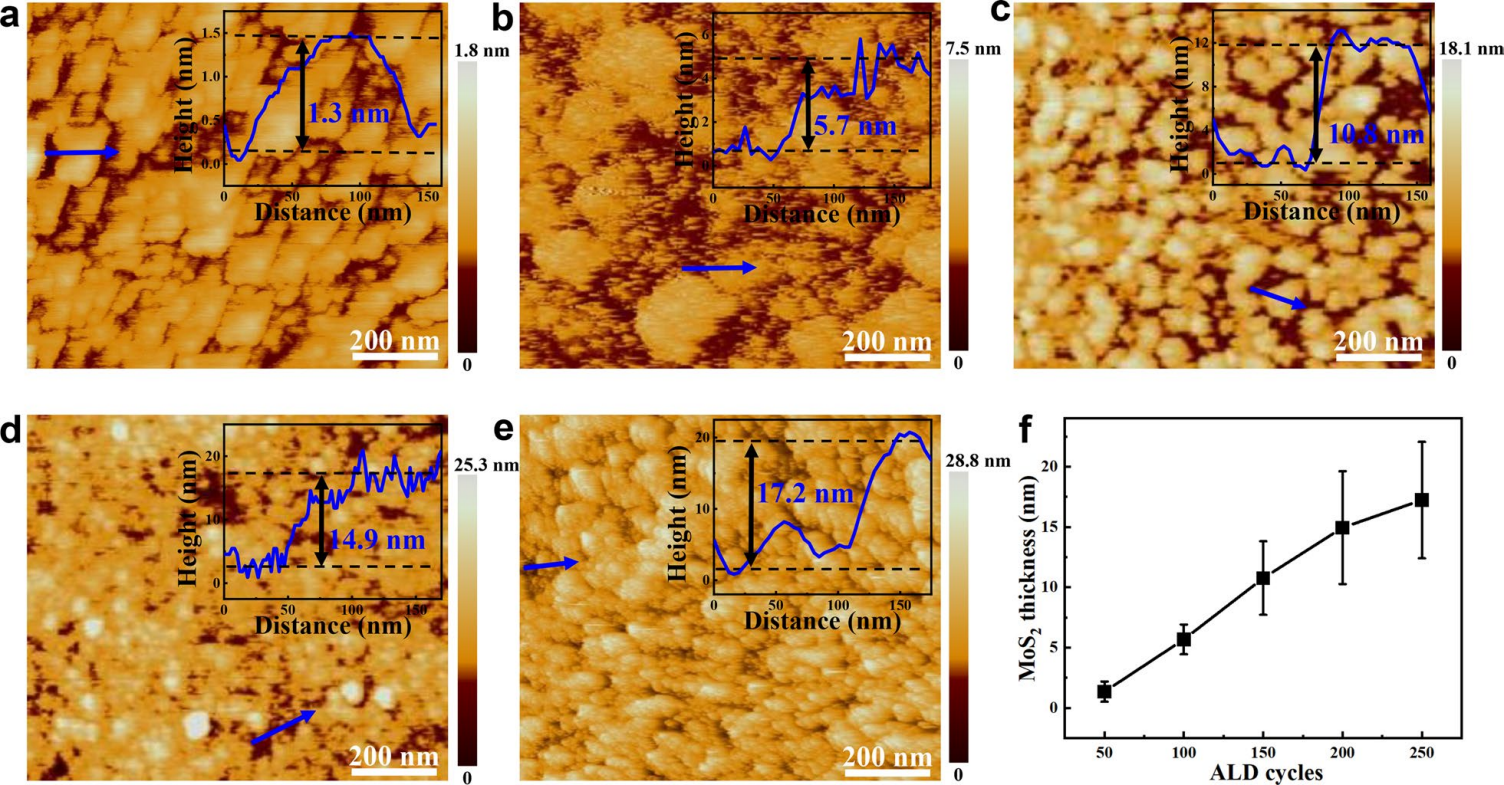
AFM images of **a** 50ALD-MoS_2_/GC, **b** 100ALD-MoS_2_/GC, **c** 150ALD-MoS_2_/GC, **d** 200ALD-MoS_2_/GC and **e** 250ALD-MoS_2_/GC. The inserted figures correspond to the height profile of AFM images in the position of blue arrows. **f** The relationship between the number of ALD cycles and the thickness of MoS_2_

The thickness of the MoS_2_ on GC is determined by measuring the height profile between the film and the substrate by atomic force microscope (AFM). From the Fig. 3a–e, as the number of ALD cycles increases (from 50 to 250), the thickness of the MoS_2_ film gradually increases (1.3, 5.7, 10.8, 14.9, and 17.2 nm, respectively). When the number of ALD cycles is 50, the thickness of MoS_2_ is about two layers, and the MoS_2_ film is not completely continuous. When the ALD cycle number reaches 250, MoS_2_ forms dense particles, which causes part of the catalytically active sites to be covered. As shown in Fig. 3f, when the number of cycles increases, the thickness of MoS_2_ increases approximately linearly, so that the thickness of MoS_2_ can be precisely controlled. The average manufacturing rate per ALD cycle is approximately 0.69 Å.

**Fig. 4:**
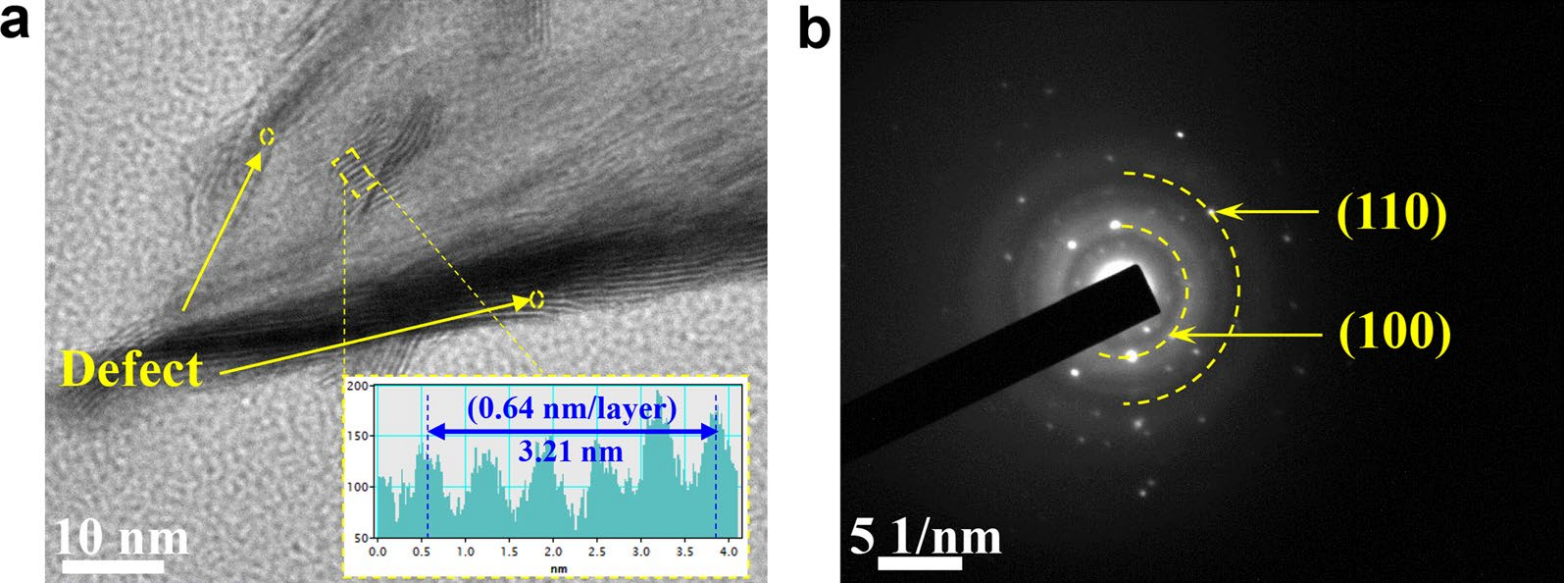
**a** HR-TEM image and **b** electron diffraction in selected area (SAED) of 200ALD-MoS_2_ film exfoliated from GC substrate

Figure 4a is the HR-TEM image of 200ALD-MoS_2_, and the lattice spacing of 0.64 nm corresponds to the (002) crystal plane spacing of MoS_2_ [31]. In addition, there are some defects on the MoS_2_ nanosheets, which is conducive to HER. In the electron diffraction in selected area (SAED), the inner layer belongs to the (100) crystal plane with 0.26 nm spacing, and the outer layer is the crystal plane with 0.16 nm (110) spacing (Fig. 4b). It can be confirmed that the crystal axis direction is the (001) direction, which indicates that the sample is composed of multiple layers of 2-D MoS_2_ nanoflakes [32].

**Fig. 5:**
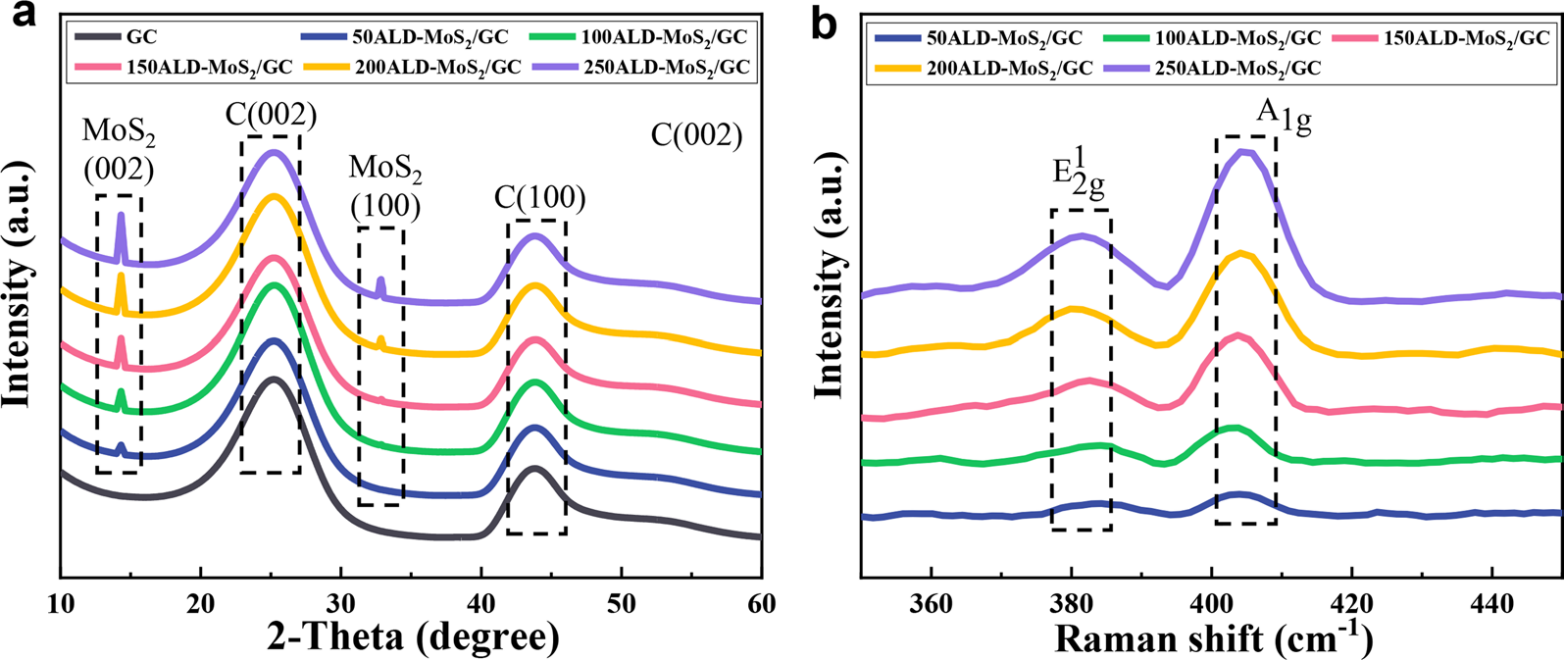
**a** XRD and **b** Raman patterns of GC and different ALD cycles MoS_2_ on GC

XRD analysis was performed on the MoS_2_ nanosheets, and the results are shown in Fig. 5a. Comparing with the standard card (JCPDScard No. 37-1492), it can be seen that the MoS_2_ films has a 2H-phase hexagonal crystal structure. The diffraction peak at 2*θ* = 14.4° is sharp and strong, which corresponds to (002) lattice plane, indicating that the MoS_2_ has a multilayer stack [33]. The diffraction peak at 32.87° corresponding to (100) plane only appears when the number of cycles is greater than 200 cycles, indicating that MoS_2_ nanosheets have out-of-plane structure [34]. Except for the carbon peak of the base GC at 16° and 43.7° corresponding to (002) and (100) planes, no other impurity peaks appeared, indicating that there are fewer impurities in the product and the reaction is relatively complete [35].

In the Raman spectrum (Fig. 5b), the vibration peaks of 382 cm^−1^ and 404 cm^−1^ are caused by the *E*_2g_^1^ and *A*_1g_ vibrational modes of MoS_2_, respectively. The *E*_2g_^1^ corresponds to the intramolecular vibration of S atoms relative to Mo atoms. The *A*_1g_ corresponds to only S atoms vibrating in the opposite direction outside the plane [34]. The difference in the peak position distance between the two peaks of MoS_2_ is sensitive to the thickness of the MoS_2_ film [36]. The position difference between the two peaks of 50ALD-MoS_2_/GC and 100ALD-MoS_2_/GC is 22.3 and 24.1 cm^−1^, respectively. It shows that MoS_2_ films are accumulating and thickening in the ALD process, which also proves that the ALD is a precise and controllable preparation method.

**Fig. 6:**
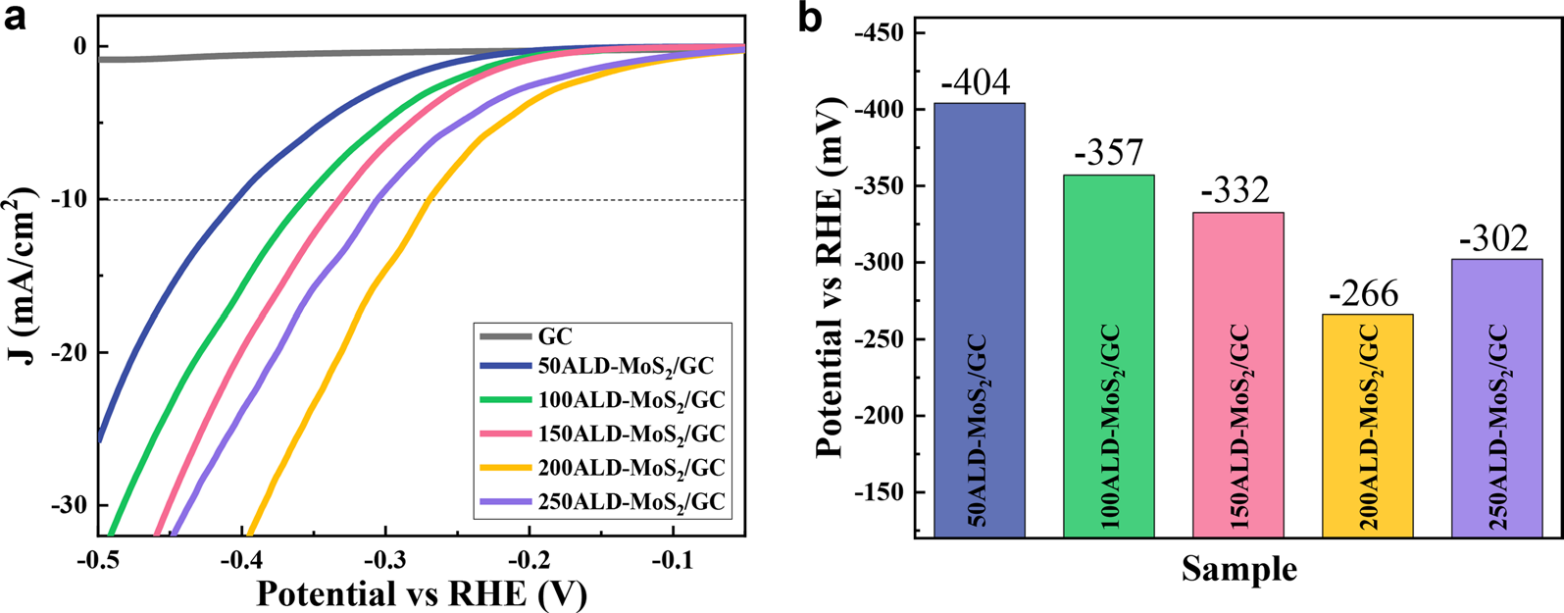
**a** Polarization curves of the various samples. **b** Potential histogram at the current density of 10 mA/cm^2^

A standard three-electrode system was used to evaluate the HER activity of MoS_2_ films with different thicknesses in a 0.5 M H_2_SO_4_ solution. Before the hydrogen evolution test, the CV test was used to pre-treat the electrode to eliminate some pollutants on the catalyst surface. It can be seen from the polarization curve (Fig. 6a) that the insignificant current density in the curve confirms that GC has almost no catalytic activity. MoS_2_ with different ALD cycles has significantly different catalytic activity, which indicates the effect of MoS_2_ with different thicknesses. Figure 6b shows the crossing point when the current density is − 10 mA/cm^2^. As the number of ALD cycles is extended from 50 to 200, the HER performance of the MoS_2_/GC gradually improves, because the amount of catalytically active MoS_2_ on the GC is increasing. When the number of ALD cycles continued to increase to 250, the catalytic performance decreased, which was due to the poor conductivity of MoS_2_ and severe aggregation resulting in a smaller number of active sites exposed. In general, the catalytic active sites on the surface of the catalyst will increase as the cycles increases and tend to be stable. However, an overly thick MoS_2_ films can cause the catalyst's conductivity to deteriorate and then increase the overpotential. Therefore, among all the catalysts, 200ALD-MoS_2_/GC shows the best HER activity, with an overpotential of 266 mV when the current density is -10 mA/cm^2^.

**Fig. 7:**
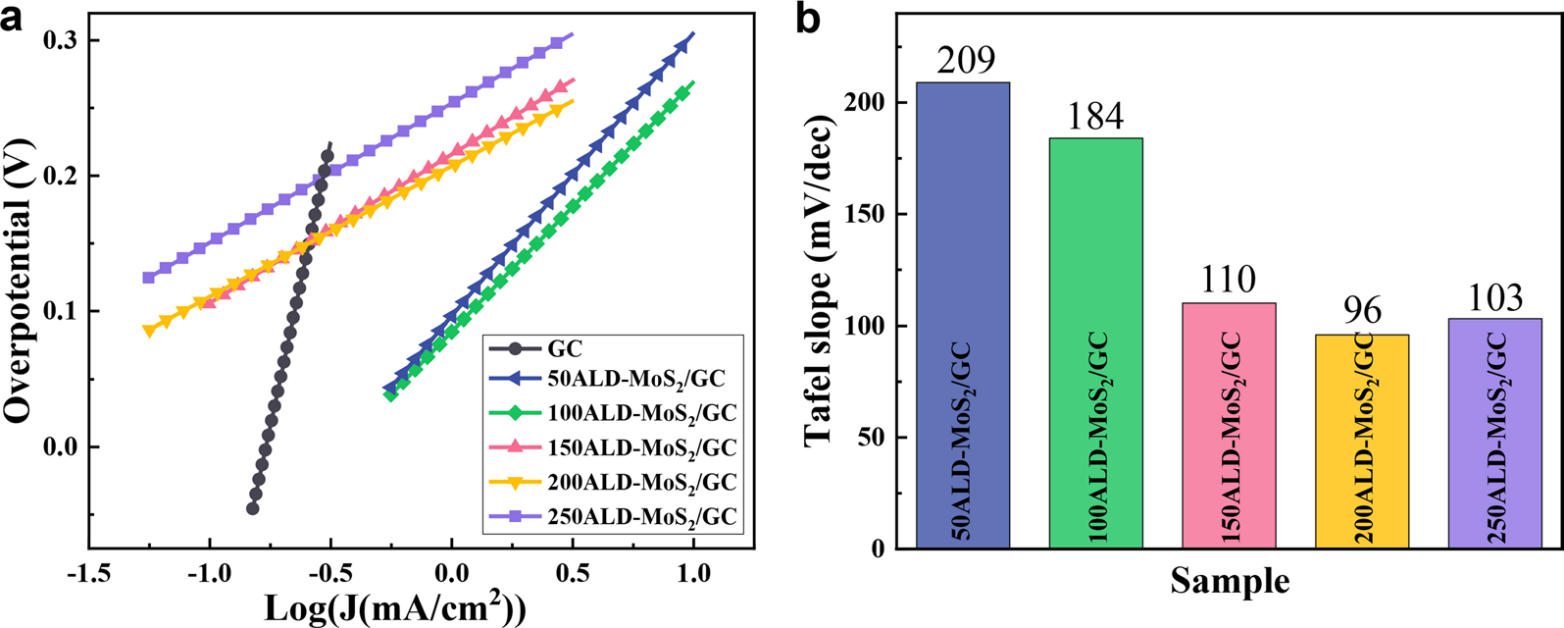
**a** Tafel plots and **b** Tafel slopes of MoS_2_ with different ALD cycles on GC

Figure 7a, b shows the Tafel curves and Tafel slopes of MoS_2_ with different ALD cycles on GC. The Tafel slope of the catalyst is negatively correlated with its electrochemical performance. The order of the Tafel slope of MoS_2_ catalysts prepared with different ALD cycles is: 209 mV/dec (50ALD-MoS_2_/GC) > 184 mV/dec (100ALD-MoS_2_/GC) > 110 mV/dec (150ALD-MoS_2_/GC) > 103 mV/dec (250ALD-MoS_2_) > 96 mV/dec (200ALD-MoS2). The 200ALD-MoS_2_/GC catalyst has the highest hydrogen evolution performance, and its electron transfer rate is also the fastest. The results also confirmed that the MoS_2_/GC HER rate control step is the Volmer reaction, that is, the generation process of adsorbed hydrogen atoms [37]. When the number of ALD cycles is 200, the amount of hydrogen adsorbed on the catalyst surface is obviously increased, which is beneficial to HER.

**Fig. 8:**
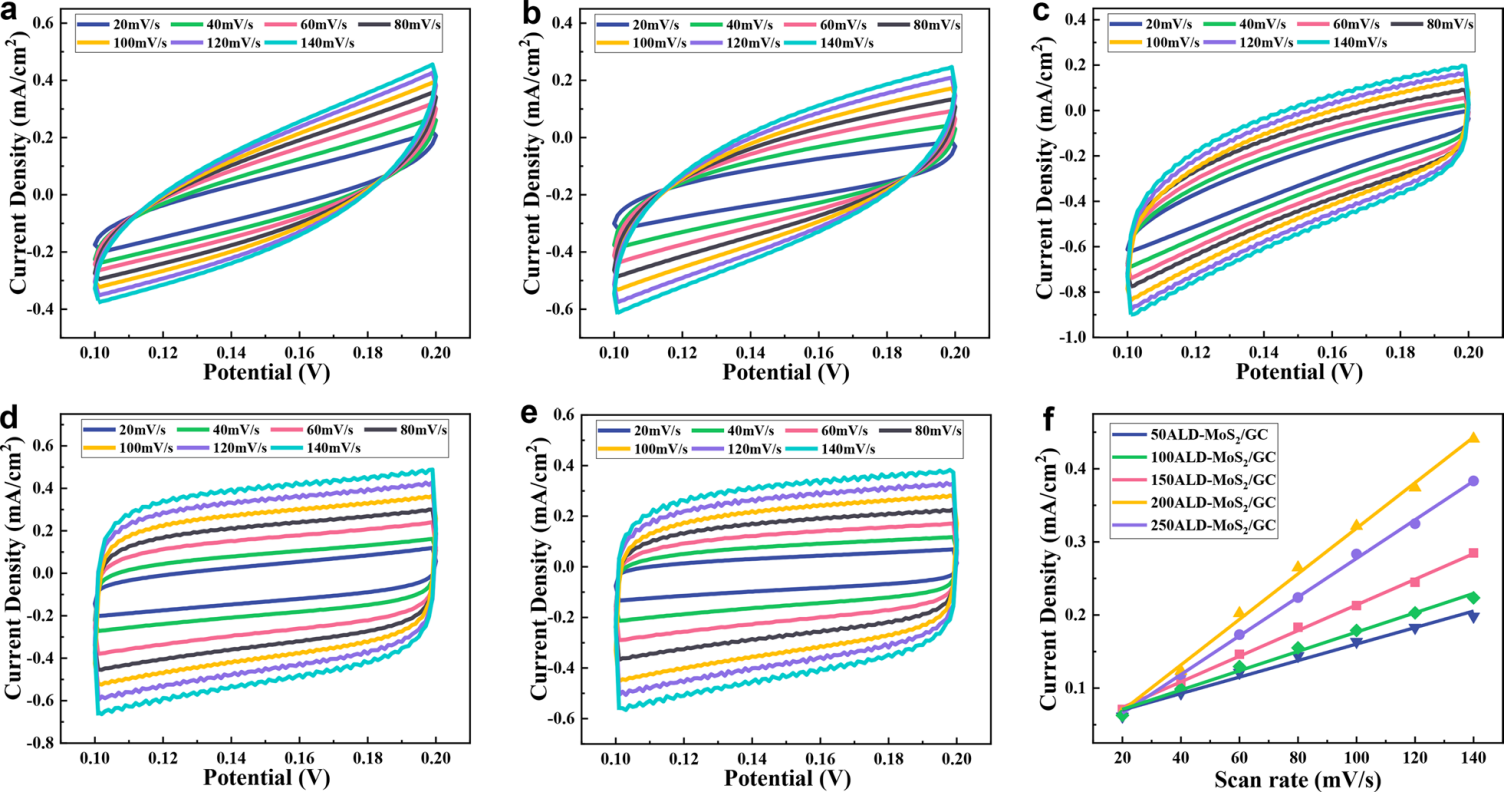
CV curves of **a** 50ALD-MoS_2_/GC, **b** 100ALD-MoS_2_/GC, **c** 150ALD-MoS_2_/GC, **d** 200ALD-MoS_2_/GC, and **e** 250ALD-MoS_2_/GC measured in 0.5 M H_2_SO_4_ in the non-Faradaic region with different scan rates from 20 to 140 mV s^−1^. **f** Differences of anodic and cathodic current densities at 0.15 V versus RHE as the functions of scan rate

The effective electrochemical active area is very important to the HER performance of the catalyst, and it is proportional to the electrochemical double capacitance (*C*_dl_). The electrochemical active area of the catalysts was compared by measuring the *C*_dl_ by CV, which provided a scientific basis for the performance comparison of the catalysts [38]. Figure 8a–e shows the CV curves of MoS_2_/GC with different thicknesses at different scan rates (20–140 mV/s). The test voltage range of CV is 0.1–0.2 V (this voltage range does not produce Faraday induced current). Subsequently, the 1/2 value of the current density difference at the intermediate potential and the scan rate are used to make a linear fitting curve diagram, and the electrochemical double-layer capacitance value of the material can be estimated from the slope of the curve. Figure 8f shows the linear relationship between current density and scan rate of MoS_2_/GC. The *C*_dl_ of 50ALD-MoS_2_/GC, 100ALD-MoS_2_/GC, 150ALD-MoS_2_/GC, 200ALD-MoS_2_/GC, and 250ALD-MoS_2_/GC are 1.13, 1.32, 1.75, 3.11, and 2.65 mF/cm^2^, respectively. Generally speaking, the active area of MoS_2_ increases with the increase of the thickness of MoS_2_, but the ECSA of 250ALD-MoS_2_/GC is lower than that of 200ALD-MoS_2_/GC, indicating that excessive MoS_2_ nanosheets would aggregate with each other to form blocks and reduce active sites.

**Fig. 9:**
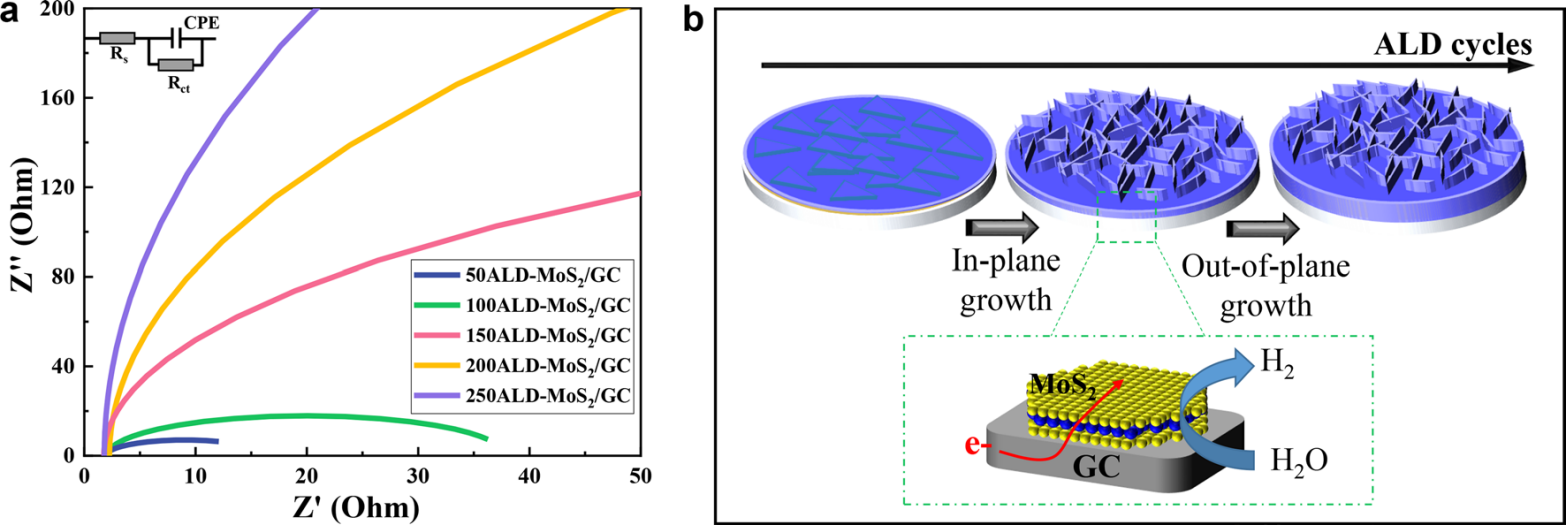
**a** Nyquist diagram of MoS_2_ with different ALD cycles. **b** Schematic showing the MoS_2_ growth and electrons transport pathway for HER

In order to deeply explore the influence of the number of ALD cycles on the HER activity, the electrochemical AC impedance method was used to conduct electrode kinetic tests on different samples, as shown in Fig. 9a. The charge transfer resistance is positively correlated with the thickness of MoS_2_, because MoS_2_ has poor conductivity. The influence of MoS_2_ thickness on HER performance was further analyzed from the ALD growth process (Fig. 9b). When the thickness of MoS_2_ is less than 3 layers, MoS_2_ grows in the vertical direction, and the triangular edge of MoS_2_ is the main catalytic site. When the thickness of MoS_2_ is greater than 3 layers, MoS_2_ growth will change from in-plane to out-of-plane, forming nanosheet-like MoS_2_. Due to the large specific surface area and many active sites of the nanosheets, it is beneficial to improve the catalytic performance. But when the thickness of MoS_2_ exceeds 15 nm, the excessive resistance will reduce the electron transfer rate, which deteriorates the electrochemical performance of the catalyst.

**Fig. 10:**
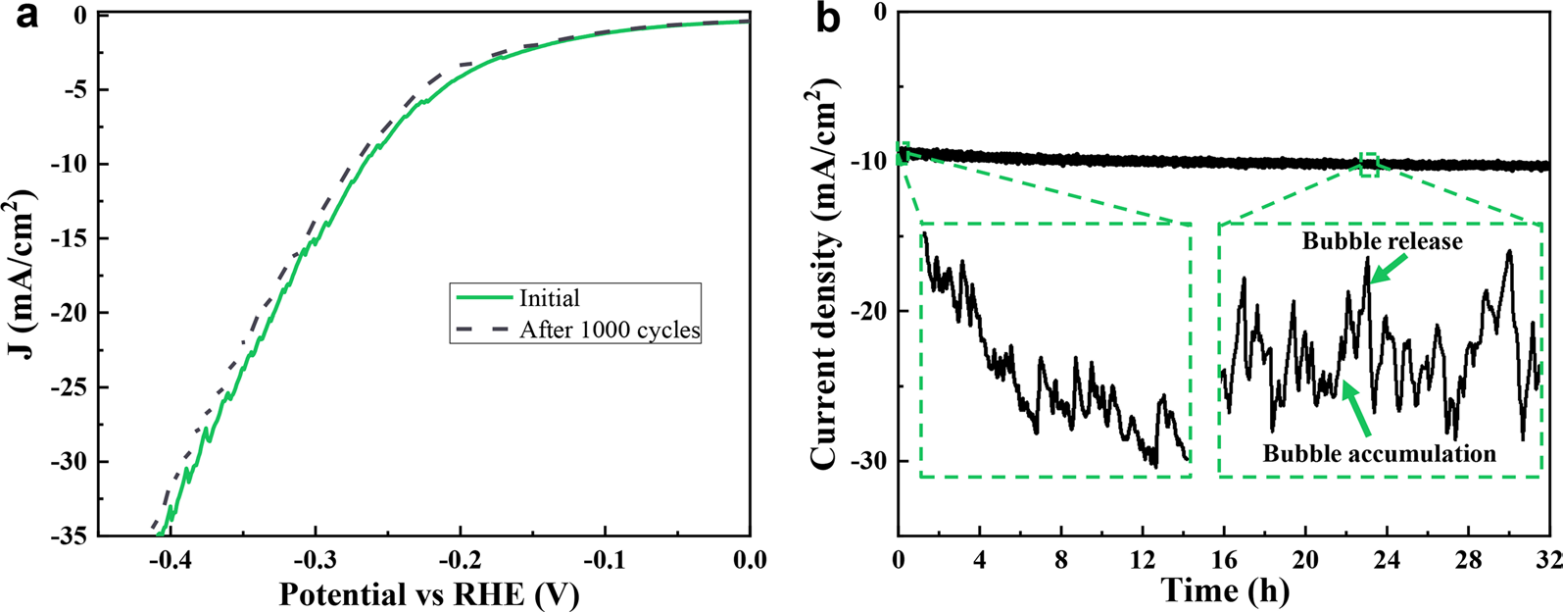
**a** Polarization curve of the electrode measured before and after 1000 CV cycles. **b** 32 h stability test at a current density of 10 mA/cm^2^

Durability and stability are also important indicators for investigating the performance of electrocatalysts [39]. In 0.5 M H_2_SO_4_ electrolyte, 200ALD-MoS_2_/GC was continuously scanned by CV, and LSV was performed after 1000 cycles. It can be seen from Fig. 10a that when the current density is -10 mA/cm^2^, the overpotential required before 1000 cycles of the catalyst is approximately 0.26 V, and the overpotential after 1000 turns is about 0.28. In addition, the activity of HER is slightly attenuated, which may be caused by a small amount of catalyst falling off the surface of the electrode. In order to further study the durability of the MoS_2_/GC catalyst, the i-t curve of the catalyst at a current density of − 10 mA/cm^2^ for 32 h was investigated. As can be seen from Fig. 10b, the potential of 200ALD-MoS_2_/GC decreased rapidly in the early stage of reaction, which was mainly because the bubbles formed by the adsorption of H^+^ in the electrolyte on the electrode surface were not desorbed in time at the early stage of reaction, so a larger overpotential was needed to maintain a fixed current density. With the extension of the reaction time, the attenuation of the curve gradually becomes flat, which is mainly caused by the close agreement between the formation rate of H_2_ bubbles on the electrode surface and the desorption rate [40]. Minor fluctuations in the *i*−*t* curve can be attributed to the generation, accumulation and release of hydrogen on the electrode surface during the reaction [41]. The results show that the MoS_2_ film manufactured by the ALD method is tightly bonded to the substrate, and has good stability during the HER. As a comparison, other studies about the electrochemical hydrogen evolution performance of MoS_2_-based nanomaterials are summarized in Table 1. It can be seen that the MoS_2_ prepared by ALD in this work has better HER performance than many MoS_2_-based composite materials, indicating that MoS_2_ with a suitable thickness can be used as an effective HER catalyst.

**Table 1:** The comparison of MoS_2_-based material for electrochemical hydrogen evolution

Catalysts	Morphology	Electrolyte	*η*_10_ (mV)	Reference
MoS_2_	Monolayer	0.5 M H_2_SO_4_	625	[42]
1 T-2H MoS_2_	Nanosheets	1 M KOH	290	[43]
MCM@MoS_2_–Ni	Nanosheets	0.5 M H_2_SO_4_	263	[44]
MoS_2_	Monolayer	0.5 M H_2_SO_4_	362	[45]
MoS_2_-CN/G	Nanosheets	0.5 M H_2_SO_4_	332	[46]
P-MoS_2_	Nanosheets	0.5 M H_2_SO_4_	250	[47]
1 T-MoS_2_	Layered structure	0.5 M H_2_SO_4_	340	[48]
MoS_2_	Nanosheets	0.5 M H_2_SO_4_	266	This work

## Conclusions

In summary, MoS_2_ films with different thicknesses were directly and accurately deposited on the GC substrate by controlling the number of cycles in the ALD process. 200ALD-MoS_2_/GC with 14.9 nm thickness shows the best HER performance, and its overpotential and Tafel slope are − 266 mV and 96 mV/dec^−1^, respectively. The catalytic activity of MoS_2_ first becomes better and then deteriorates with the increase of its thickness. Because the dense MoS_2_ nanosheets aggregate with each other to reduce the active sites and increase the resistance. In addition, the MoS_2_ films prepared by ALD are firmly bonded to the substrate, showing excellent stability. This work reveals that the appropriate thickness of MoS_2_ films is beneficial to the optimization of electrocatalytic performance, which has great inspiration for MoS_2_ to replace noble metal catalysts for hydrogen evolution.

## Data Availability

All data are fully available without restriction.

## Abbreviations

MoS_2_: Molybdenum disulfide
HER: Hydrogen evolution reaction;
GC: Glassy carbon
ALD: Atomic layer deposition
2-D: Two-dimensional
CVD: Chemical vapor deposition
SEM: Scanning electron microscopy
XRD: X-ray diffraction
HR-TEM: High resolution-transmission electron microscope
SAED: Selected area electron diffraction
LSV: Linear scanning voltammetry
CV: Cyclic voltammetry
EIS: Electrochemical impedance spectroscopy
*C*_dl_: The double-layer capacitance
RHE: Reversible hydrogen electrode
